# Overexpression of regulatory T cells in patients with unexplained recurrent pregnancy loss: friend or foe?

**DOI:** 10.3389/fmed.2023.1244424

**Published:** 2024-01-04

**Authors:** Peng-cheng Liu, Jian-bin Li, Yi-ping Huang, Min Zhang, Shu-jiao Yu, Rui Wu

**Affiliations:** Department of Rheumatology, The First Affiliated Hospital, Jiangxi Medical College, Nanchang University, Nanchang, China

**Keywords:** recurrent pregnancy loss, pregnancy outcome, regulatory T cells, U-shaped association, biomarker

## Abstract

**Background:**

This study aimed to investigate the role of regulatory T cells in patients with unexplained recurrent pregnancy loss (URPL).

**Methods:**

We retrospectively analyzed 136 women who had experienced two or more miscarriages before 24 weeks of gestation for no obvious reason from May 2018 to October 2021. The basic clinical data of the patients and expression of lymphocyte subsets such as regulatory T cells (Tregs) and natural killer cells (NKs) by flow cytometry were collected to explore the risk factors of pregnancy outcome in URPL patients.

**Results:**

A total of 136 URPL patients were enrolled in this study. Eventually, 50 patients attained clinical pregnancy. The median age was 31.8 ± 4.6 years in patients with clinical pregnancy. The univariate and multivariate logistic regression analyses indicated that Tregs was associated with the pregnancy outcomes of patients with URPL (odds ratio 0.63, 95% confidence interval 0.50–0.80). More importantly, a U-shaped association was found between Tregs and pregnancy outcome (*p* < 0.001), with either higher or lower Tregs levels adversely affecting pregnancy outcome.

**Conclusion:**

Tregs levels that are either too high or too low can harm pregnancy outcomes. It was expected to be a very promising quantitative biomarker for predicting pregnancy outcomes in URPL patients.

## Introduction

Recurrent pregnancy loss (RPL) is defined as two or more miscarriages with the same sexual partner within 24 weeks of gestation, with a prevalence ranging from 1%–5% (1). In approximately 50% of patients with RPL, the underlying cause remains unknown, and 80% of unexplained miscarriages are closely related to immune factors (2). Due to poor pregnancy outcomes, patients with RPL often face great psychological and social pressure.

Among the known causes, immune factors, thrombophilia, anatomical abnormalities of the uterus and endocrine abnormalities are the 4 most important causes (3). Immunological factors are divided into autoimmunity and alloimmunity. Autoimmunity typically involves the presence of autoantibodies, resulting in tissue and organ damage and pregnancy loss. Clinical diagnosis involves testing for autoantibodies. Alloimmunity refers to the inability of the body to develop appropriate immune tolerance over a certain period, resulting in maternal rejection of the embryo and subsequent miscarriage (4). Because of the lack of a specific test, alloimmunization is still a controversial study, although there is growing evidence to support the possibility that RPL of unknown origin may be related to alloimmunization (5). However, there is still no clear and effective method to predict recurrent pregnancy loss of unknown origin.

At the placenta, natural killer cells (NKs) are the most prevalent form of lymphocyte. Placental NKs exhibit low cytotoxicity and can aid in the formation of blood vessels, in contrast to blood NK (6). Nevertheless, it has been noted that increased NKs activity is detrimental to pregnancy and associated with unexplained recurrent pregnancy loss (URPL) (7). Another crucial lymphocyte in the placenta is Tregs, known for their immunosuppressive role in preventing activated NKs and CD8^+^ T cells from causing harm (8). Tregs are generally amplified at the maternal-fetal interface and in peripheral blood after delivery (9). However, some studies had shown that URPL patients did not experience this amplification (10, 11). Although the majority of URPL patients seek medical attention when they are not pregnant, abnormal immune activation or a deficiency of immune cells during pregnancy appear to be connected to URPL. It’s still unclear whether the aberrant immunological state may be identified before pregnancy.

Indeed, some studies (12, 13) show that low levels of Tregs can harm pregnancy outcomes in URPL patients. However, the effect of high levels of Tregs on pregnancy outcomes in URPL patients is easily neglected. In our study, restrictive cubic spline (RCS) flexible modeling was used to explore the potential nonlinear correlation between the Tregs and the pregnancy outcomes in URPL.

## Methods

### Study design

**Figure 1:**
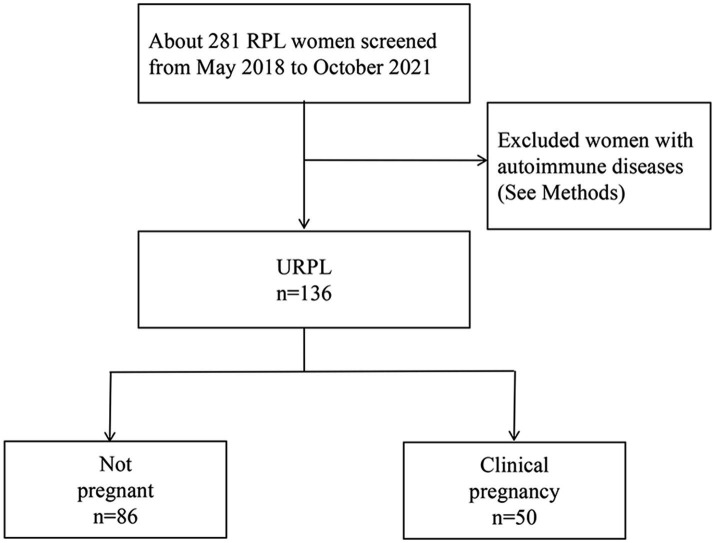
Flow diagram for study population. RPL, recurrent pregnancy loss; URPL, unexplained recurrent pregnancy loss.

We evaluated 136 patients in the database of the First Affiliated Hospital of Nanchang University who had experienced two or more unexplained miscarriages before 24 weeks of gestation from May 2018 to October 2021. Among them, 50 patients (the pregnancy group) had successful pregnancies, while 86 patients (the control group) experienced pregnancy losses. We compared basic clinical data, laboratory test results, and changes in lymphocyte subpopulations between the two groups. Additionally, we analyzed the risk factors for pregnancy outcomes in patients with URPL (a flow diagram of the study design is depicted in Figure 1).

The following inclusion and diagnostic criteria and exclusion criteria were used for all enrolled patients. The inclusion criteria for patients were as follows: all patients met the diagnostic criteria for RPL as defined by the American Society for Reproductive Medicine (ASRM) (1); normal spousal semen examination; absence of abnormal karyotype in both spouses; the following exclusion criteria were also met: autoimmune diseases with positive autoantibodies; combined organic pelvic genital tract disease; cervical secretions with detectable reproductive tract infection; combined blood hypercoagulable state; combined ovarian tumors.

### Detection

Fasting peripheral venous blood samples were collected from all patients with RPL and immediately sent to the Department of Laboratory Medicine of the First Affiliated Hospital of Nanchang University for testing. Lymphocyte subsets were detected by flow cytometry: specifically, Tregs (CD3^+^ CD4^+^ CD25^+^ CD127^dim/−^), CD4^+^ T cells (CD3^+^ CD4^+^) ratio, CD8^+^ T cells (CD3^+^ CD8^+^) ratio, B cells (CD3^−^ CD19^+^) ratio, natural killer cells (NK cells; CD3^−^ CD56^+^ CD16^+^) ratio, while recording their synchronized lymphocyte absolute results, and further calculating the absolute cell counts of each lymphocyte subpopulation.

Specific assay method: 50 μL of peripheral venous blood was collected, anticoagulated, and placed in an absolute counting tube, 15 μL of 6-color lymphocyte subpopulation antibody was added and mixed, incubated for 15 min, then 450 μL of hemolytic agent for flow cytometry was added, mixed thoroughly, and placed for 10 min in the dark, followed by centrifugation at 2,500 r/min (centrifugation radius 10 cm) for 6 min, the supernatant was removed, and 100 μL of PBS was added, it was done twice, and then put on the flow cytometer. The lymphocyte subpopulations in the blood specimens were detected using a BD FACSCANTO II flow cytometer (BD, United States). For regulatory T cells assay, 100 μL of whole blood anticoagulated with sodium heparin was taken, labeled anti-human CD4-FITC, and CD25-APC antibodies were added, incubated for 20 min at 4°C and protected from light, erythrocyte lysate was added, left at room temperature for 10 min, rinsed twice with PBS, add membrane breaking liquid and placed at 4°C in the dark for 1 h, rinsed twice with PBS, and detected by flow cytometry.

### Nonlinear analysis

Nonlinear analysis is a versatile mathematical and statistical approach crucial for investigating relationships and phenomena that deviate from linear patterns. In comparison with generalized linear models, Nonlinear analysis can unveil the intricacies of real-world data, providing a comprehensive understanding of dynamic processes, and aiding in predictions, modeling, and decision-making. Especially in the context of generalized linear models, it is often assumed that the relationship between outcomes and variables is linear, yet this frequently overlooks the potential presence of nonlinear relationships between outcomes and variables. Therefore, in this study, we employed nonlinear analysis to investigate the underlying nonlinear relationship between the Tregs and the pregnancy outcome in URPL.

### Statistical analysis

SPSS version 26.0 (IBM, Armonk, NY, United States) was used for statistical analysis. Continuous variables were described as the mean ± standard deviation for normally distributed data. Categorical variables were described as frequencies and percentages. For normally distributed continuous data, differences between the two groups were analyzed using an independent samples *t*-test. Categorical variables were evaluated using either the chi-square test or Fisher’s exact test to determine differences between the two groups. Pearson’s test was employed for the correlation analysis. Univariate and multivariate logistic regression analysis was applied for the analysis of the risk factors of pregnancy outcomes in URPL patients. Restrictive cubic spline (RCS) flexible modeling was used to explore the potential nonlinear correlation between the Tregs and the pregnancy outcome in URPL. All statistical tests were two-tailed, and *p* < 0.05 was considered statistically significant.

## Results

### Baseline characteristics

A total of 136 patients with URPL were included in this study, and the mean age of those with URPL was 31.7 ± 4.6 years. Among them, 50 patients (36.8%) had successful pregnancies; 86 patients (63.2%) had pregnancy losses. According to the report provided by the Clinical Laboratory Department of the First Affiliated Hospital of Nanchang University, the normal reference range for Tregs (as a proportion of total CD4^+^ cells) is 4 to 9. In our study, we observed that among 50 patients with successful pregnancies, only 8 had Tregs in the abnormal range, while among the 86 patients with pregnancy loss, 47 had Tregs in the abnormal range. Meanwhile, we found significant differences in Tregs and NK cells between the two groups (*p* < 0.05). The baseline characteristics and outcomes are shown in Table 1.

**Table 1:** Baseline characteristics of all qualified participants.

	Total *n* = 136	Successful pregnancies *n* = 50	Pregnancy loss *n* = 86	*p*-value
Age, years	31.7 ± 4.6	31.8 ± 4.6	31.7 ± 4.6	0.939
Number of miscarriages				0.795
≥3	39 (28.7%)^a^	15 (30.0%)^a^	24 (27.9%)^a^	
<3	97 (71.3%)^a^	35 (70.0%)^a^	62 (72.1%)^a^	
Tregs (abnormal)^c^	55 (40.4%)^a^	8 (16.0%)^a^	47 (54.7%)^a^	**<0.001**
<4	45 (33.1%)^a^	3 (6.0%)^b^	42 (48.9%)^a^	
>9	10 (7.3%)^a^	5 (10.0%)^a^	5 (5.8%)^a^	
CD3^+^ cells	1245.5 ± 380.7	1309.3 ± 433.1	1218.6 ± 356.2	0.276
CD3^+^ CD4^+^ cells	661.0 ± 209.9	711.9 ± 246.3	639.5 ± 190.4	0.114
CD3^+^ CD8^+^ cells	464.2 ± 199.8	490.5 ± 201.3	453.1 ± 199.5	0.393
CD4/CD8 ratio	1.6 ± 0.6	1.5 ± 0.5	1.6 ± 0.6	0.811
NK cells	291.4 ± 175.5	234.5 ± 166.3	324.5 ± 173.1	**0.004**
B cells	215.3 ± 95.6	214.2 ± 71.9	215.8 ± 104.3	0.94

Tregs, regulatory T cells; bold values are statistically significant (*p* < 0.05).

a The categorical data were described as frequencies (percentages), and the differences between the two groups were tested by the chi-square test.

b For categorical data with counts less than 5, differences between the two groups were tested by the Fisher’s exact test.

c According to the report provided by the Clinical Laboratory Department of the First Affiliated Hospital of Nanchang University, the normal reference range for Tregs (as a proportion of total CD4^+^cells) is 4 to 9.

### Correlation analysis between other lymphocyte subsets and Tregs

**Figure 2:**
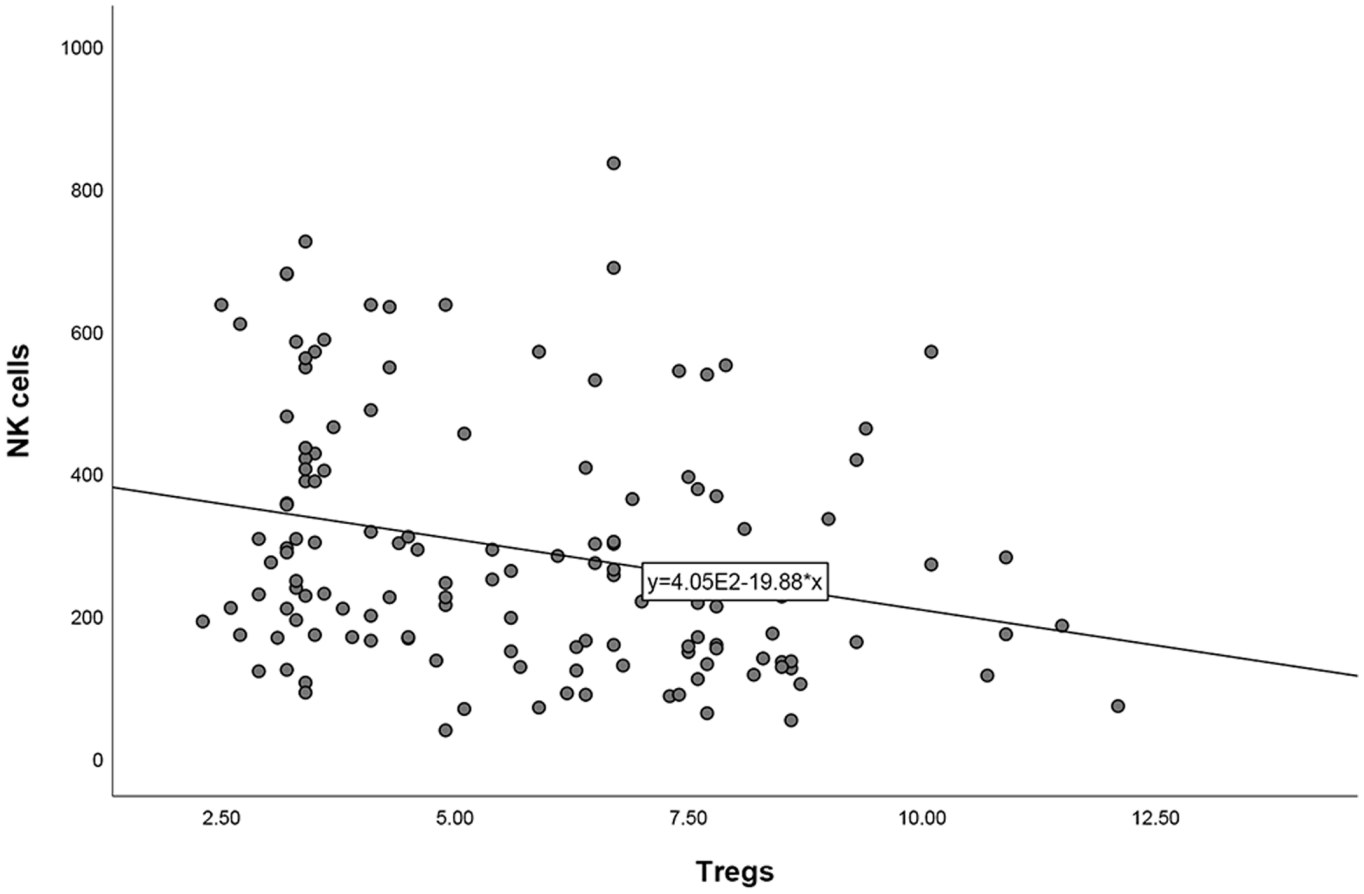
Correlation analysis between Tregs and NK cells.

After correlation analyses between other lymphocyte subsets and Tregs in patients with URPL, it was found that Tregs was negatively correlated with NK cells (*r* = −0.262, *p* = 0.002). No other correlation analyses were found significant between other lymphocyte subsets and Tregs (Figure 2).

### Univariate and multivariate logistic regression analyses for pregnancy outcome

The univariate logistic regression analyses suggested that Tregs and NK cells might be associated with pregnancy outcomes (Table 2). After multivariate logistic regression analysis, the result indicated that Tregs (OR = 0.63, *p* < 0.001) were associated with the pregnancy outcomes of patients with URPL (Table 3).

**Table 2:** Univariate regression for effect of Tregs on pregnancy outcome.

Parameters	OR	95% CI	*p*-value
Tregs	0.60	0.49–0.73	**<0.001**
CD3^+^ cells	1.00	1.00–1.00	0.275
CD3^+^ CD4^+^ cells	1.00	1.00–1.00	0.118
CD3^+^ CD8^+^ cells	1.00	1.00–1.00	0.392
CD4/CD8 ratio	1.10	0.51–2.38	0.809
NK cells	1.00	1.00–1.01	**0.005**
B cells	1.00	1.00–1.00	0.939

Tregs, R regulatory T cells; OR, odds ratio; 95% CI, 95% confidence interval. Bold values are statistically significant (*p* < 0.05). Outcome variable: successful pregnancies. Two reference groups: successful pregnancies group and pregnancy loss group.

**Table 3:** Multivariate regression for effect of Tregs on pregnancy outcome.

Parameters	OR	95% CI	*p*-value
Age	0.94	0.83–1.06	0.342
Number of miscarriages	0.65	0.21–2.02	0.455
Tregs	0.63	0.50–0.80	**<0.001**
CD3+ cells	1.00	1.00–1.01	0.423
CD3^+^ CD4^+^ cells	0.99	0.98–1.00	0.093
CD3^+^ CD8^+^ cells	1.00	0.99–1.01	0.707
CD4/CD8 ratio	2.18	0.24–19.80	0.488
NK cells	1.00	0.99–1.00	0.330
B cells	1.01	1.00–1.01	0.059

Tregs, R regulatory T cells; OR, odds ratio; 95% CI, 95% confidence interval. Bold values are statistically significant (*p* < 0.05). Outcome variable: successful pregnancies. Two reference groups: successful pregnancies group and pregnancy loss group.

### Nonlinear relationship between Tregs and the pregnancy outcomes

**Figure 3:**
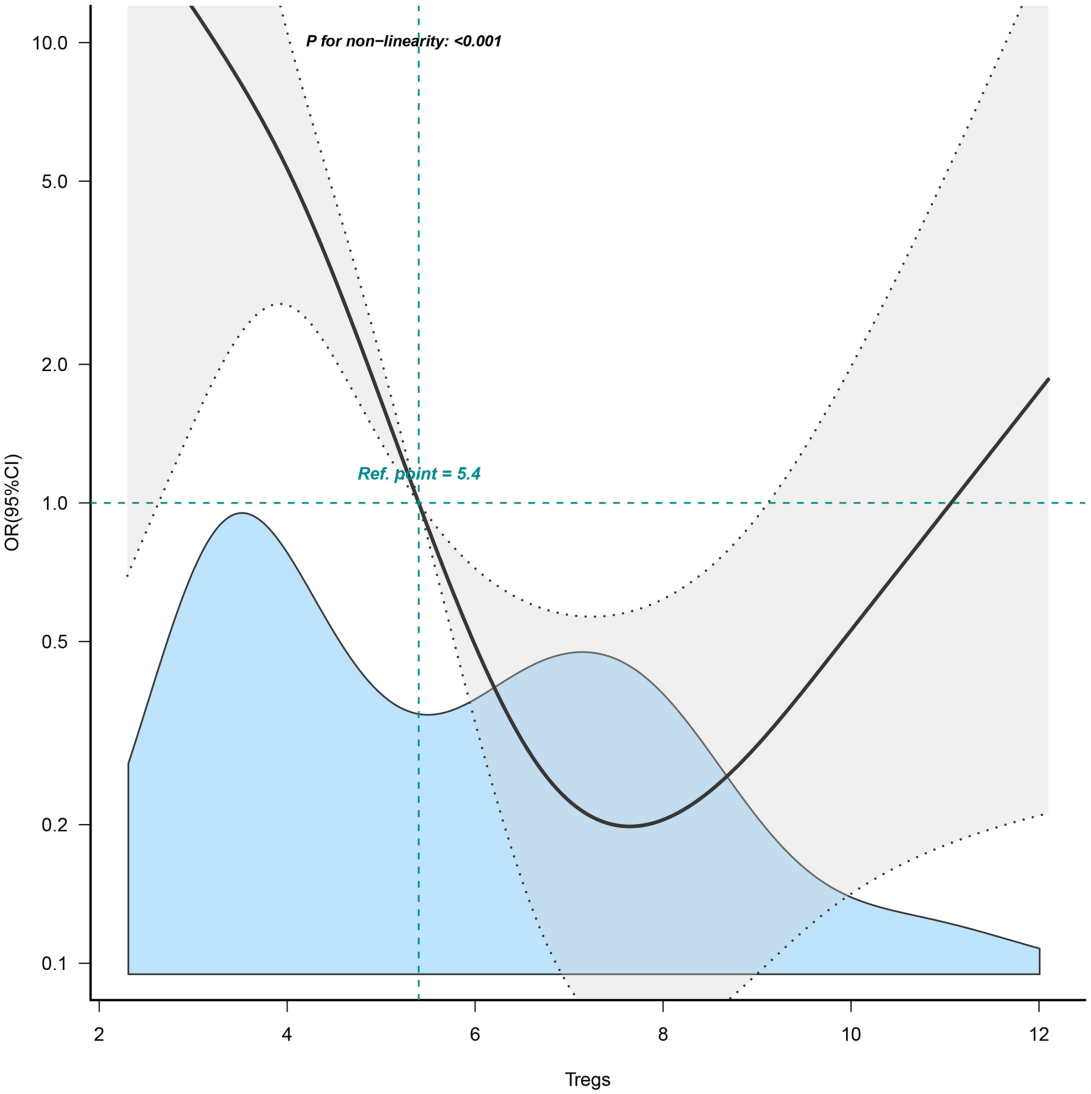
Nonlinear relationship between Tregs and the pregnancy outcomes of patients with URPL.

In Figure 3, a nonlinear relationship was observed for the first time between Tregs and the pregnancy outcomes of patients with URPL (*p* < 0.001). The results of the nonlinear analysis showed a U-shaped distribution of the effect of Tregs on pregnancy outcome, with either higher or lower Treg cell levels adversely affecting pregnancy outcome.

## Discussion

In recent years, the number of patients with URPL has gradually increased, and women with a history of multiple miscarriages are under great physical and psychological stress. Effective treatment options to regulate immune function to prevent miscarriage are under intensive research. The evolving understanding of reproductive immunity has linked the etiology of recurrent miscarriage to immune disorders. It is now acknowledged that approximately 50%–60% of recurrent miscarriages are associated with immunological factors (2). Of these, around 2/3 fall into the category of alloimmune-type recurrent miscarriage. This subgroup comprises patients with URPL experiencing an alloimmune state characterized by an imbalance at the maternal-fetal interface, leading to intrinsic or acquired immune disorders. Examples include abnormalities in NK cells, Th1/Th2 cell imbalance, imbalances in the Th17 cell subpopulation and Tregs, and other immune disorders. Importantly, these disorders do not cause systemic organ tissue damage in pregnant women (14). It is important to explore the impact of immune disorders, immunotherapy, and some other indicators on pregnancy outcomes in these patients to clinically guide the treatment of URPL patients.

Regulatory T cells, also known as Tregs, are a subset of CD4^+^ T helper cells that suppress the immune system (15). Through interactions with target cells, secretion of anti-inflammatory cytokines like IL-10, competition with CTL cell inducer IL-2 for occupancy, and other mechanisms, Tregs effectively control local inflammatory responses (16). Thus, it is a cell type with potent immunosuppressive activity. Antigenic stimulus is necessary for Treg activation, just like it is for other T cells. A study discovered that following fetal implantation, Tregs in the decidua and peripheral blood rapidly grew as a result of embryonic antigen stimulation (17), but a different study indicated that URPL patients did not experience this increase (10, 11). In a DBA × CBA abortion model, adoptive infusion of Tregs from pregnant mice saved embryos from resorption (18). According to all available research, the failure of Tregs to expand throughout pregnancy may be connected to the phenomena of recurrent miscarriage, which shows that Tregs are essential for maintaining immunological tolerance at the maternal-fetal interface.

The quantity and function of Tregs may undergo changes during pregnancy to accommodate the maternal immune system’s tolerance towards the fetus. During early pregnancy (first to third month), Treg cell levels are relatively low. This is primarily to avoid excessive suppression of immune responses and to maintain defense against pathogens (19). During this period, cells in the placenta produce a series of molecular signals to suppress the maternal immune system, thus protecting the embryo’s survival. As pregnancy progresses into the mid-term (fourth to sixth month), Tregs gradually increase. This increase may help in suppressing autoimmune responses, reducing immune attacks on the fetus, and maintaining normal fetal development (20). By late pregnancy (seventh to ninth month), the levels of Tregs further increase. This increase may contribute to protecting the fetus from immune-related complications, such as intrauterine growth restriction and preterm birth. In our study, all Tregs measurements of enrolled patients were conducted within 1–2 weeks prior to the next pregnancy. This avoids the Tregs detection discrepancies caused by different stages of pregnancy.

It is noteworthy that previous studies have suggested differences in the distribution of Tregs between the decidua and peripheral blood (21, 22). The study by Tilburgs et al. (23) revealed Tregs to be much more abundant in decidual samples compared to peripheral blood samples in pregnant patients. Moreover, their findings demonstrated a markedly elevated proportion of Tregs in the decidua parietalis in contrast to the decidua basalis. Regrettably, constrained by technical limitations, this study conducted detection of Tregs in the peripheral blood. Subsequent assessments of Tregs in the placenta are essential to further validate the findings of this study.

Indeed, many researchers have reported that women with URPL have lower Tregs levels than normal patients (24, 25). Up to now, numerous results generally suggest that lower Tregs level is associated with a higher risk of miscarriage in women with URPL (12, 13, 26). However, our study revealed for the first time that either lower or higher Tregs levels increase the risk of miscarriage in women with URPL. We found the effect of Tregs on pregnancy outcome has a U-shaped distribution, which is different from the results of many previous studies. On the one hand, the reduced number of blood Tregs may be a manifestation of impaired Treg amplification. Two types of Tregs are produced in different ways. Firstly, the production of natural Treg (nTreg) is thymus dependent (27, 28). Secondly, under the induction of TGF-β and other cytokines, naive T cells can differentiate into Tregs that show high expression of Foxp3 (29, 30). Following semen and embryonic antigen stimulation, Tregs expand rapidly during the first trimester of pregnancy. In fact, this expansion of Tregs is important in maintaining immune tolerance, and therefore failure of expansion may be a cause of miscarriage.

On the other hand, Tregs also have many subtypes (31), among which we speculate that both Tregs subtypes with immunosuppressive effects and Tregs subtypes with pro-inflammatory effects exist. Therefore, when Tregs levels are too high, immune tolerance at the maternal-fetal interface can also be influenced, leading to adverse pregnancy outcomes. A study by Professor Takuro Saito indicated that Tregs can be divided into three subpopulations based on FOXP3 and CD45RA expression levels. After being stimulated by an antigen, Fraction I (Fr-I; FOXP3^lo^CD45RA^+^; also known as naive Treg (nTreg)) cells differentiate into Fraction II (FOXP3^hi^CD45RA^+^; also known as effector Treg (eTreg)) cells, which are terminally differentiated, highly suppressive, and functionally stable. Fr-III (FOXP3^lo^CD45RA^−^) cells, in contrast, lack suppressive function and are capable of secreting pro-inflammatory cytokines (32). The above findings provided us with a strong support for the analysis of the results.

We acknowledge the potential limitations of our study. First, although our study revealed for the first time that either lower or higher Tregs levels increase the risk of miscarriage in women with URPL, we did not deeply explore the specific reasons. We will conduct in-depth analyses on different Tregs subpopulations in subsequent studies to clarify the specific mechanisms of the effect of lower or higher Tregs levels on pregnancy outcomes. Second, this study is a preliminary exploratory study, and we next hope to conduct a prospective, large sample study to further confirm the validity of Tregs level in predicting the risk of miscarriage in women with URPL.

## Conclusion

We revealed for the first time a deeper nonlinear relationship between Tregs and the pregnancy outcomes of patients with URPL. More importantly, Tregs was expected to be a very promising quantitative biomarker for predicting pregnancy outcomes in URPL patients.

## Data availability statement

The raw data supporting the conclusions of this article will be made available by the authors, without undue reservation.

## Ethics statement

The studies involving humans were approved by the Ethics Committee of the First Affiliated Hospital of Nanchang University. The studies were conducted in accordance with the local legislation and institutional requirements. The ethics committee/institutional review board waived the requirement of written informed consent for participation from the participants or the participants’ legal guardians/next of kin because the requirement for informed consent from the study participants was waived in accordance with local legislation and institutional requirements.

## Author contributions

P-cL and RW: study design and drafting of the manuscript. J-bL and Y-pH: data collection and interpretation. P-cL and MZ: data analysis. J-bL, Y-pH, and S-jY: modification and polishing. All authors contributed to the article and approved the submitted version.
